# Chronic activation of the D156A point mutant of Channelrhodopsin-2 signals apoptotic cell death: the good and the bad

**DOI:** 10.1038/cddis.2016.351

**Published:** 2016-11-03

**Authors:** Michael Perny, Lukas Muri, Heather Dawson, Sonja Kleinlogel

**Affiliations:** 1 Institute for Physiology, University of Bern, Bern 3012 Switzerland; 2 Institute of Pathology, Clinical Pathology Division, University of Bern, Bern 3010 Switzerland

## Abstract

Channelrhodopsin-2 (ChR2) has become a celebrated research tool and is considered a promising potential therapeutic for neurological disorders. While making its way into the clinic, concerns about the safety of chronic ChR2 activation have emerged; in particular as the high-intensity blue light illumination needed for ChR2 activation may be phototoxic. Here we set out to quantify for the first time the cytotoxic effects of chronic ChR2 activation. We studied the safety of prolonged illumination on ChR2(D156A)-expressing human melanoma cells as cancer cells are notorious for their resistance to killing. Three days of illumination eradicated the entire ChR2(D156A)-expressing cell population through mitochondria-mediated apoptosis, whereas blue light activation of non-expressing control cells did not significantly compromise cell viability. In other words, chronic high-intensity blue light illumination alone is not phototoxic, but prolonged ChR2 activation induces mitochondria-mediated apoptosis. The results are alarming for gain-of-function translational neurological studies but open the possibility to optogenetically manipulate the viability of non-excitable cells, such as cancer cells. In a second set of experiments we therefore evaluated the feasibility to put melanoma cell proliferation and apoptosis under the control of light by transdermally illuminating *in vivo* melanoma xenografts expressing ChR2(D156A). We show clear proof of principle that light treatment inhibits and even reverses tumor growth, rendering ChR2s potential tools for targeted light-therapy of cancers.

In the last decade optogenetics has revolutionized the neurosciences enabling neuroscientists to link neural network activity with behavior and disease. Latter in particular fostered the development of optogenetic treatment protocols for potential use in the clinic. A channelrhodopsin-2 (ChR2)-based therapy to recover vision in the blind has recently been approved for clinical trials (NCT02556736) and optogenetic deep brain stimulation for motor and mood disorders such as Parkinson's and depression are currently under active investigation. The rapid development of ChR2 as a therapeutic tool has raised concerns about the safety of the required chronic high-intensity blue light activation and has spurred the development of more light-sensitive (CatCh,^1^ ChR2(D156A),^2^ Opto-mGluR6^3^) and red-shifted (VChR1,^4^ ReaChR,^5^ Chrimson^6^) ChR2 variants, as longer wavelengths are less harmful.^7^ Also the potentially non-physiological activation mediated by ChR2s through continuous strong depolarization combined with Ca^2+^ influx^1, 8^ has raised concerns and alternative tools have been developed that light-activate the native signaling pathways of target cells.^3^^,^^8^^,^^9^

Here we quantified for the first time the blue light and the ChR2-induced cytotoxicities. To rigorously probe for the induced changes in cell viability we used a human melanoma cell line, as cancer cells are renowned for their resistance to killing.^10, 11 and 1112^ We chose the light-sensitive slow ChR2(D156A) point mutant^2^ as optogenetic actuator and showed that 3 days of continuous pulsed illumination killed all ChR2(D156A)-expressing melanoma cells by mitochondria-induced apoptosis. However, illumination alone did not have any significant effects on cell viability, indicating that phototoxicity is not of primary concern, but instead it appears to be the chronic depolarization, potentially combined with constant Ca^2+^ inflow into the cytoplasm mediated through ChR2(D156A) that cause the cytotoxic effects.

The discovery of light-induced apoptotic signaling in cancer cells highlights an opportunity for targeted cancer cell therapy. In a second set of experiments we give proof-of-principle that optogenetic transdermal light treatment of melanoma xenografts in mice terminates tumor growth. Sparing healthy tissue from therapy exposure is a critical challenge in the treatment of cancer that could be overcome in an optogenetic therapy by localized photoactivation.

## Results

To quantify the potential cytotoxic effects of chronic ChR2 activation we employed the 100-fold more light-sensitive D156A mutant of ChR2, which possesses the longest channel open lifetime so far reported (*τ*_*off*_>150 s), mediating sustained channel opening under pulsed blue light stimulation.^2^ Long-term pulsed illumination is experimentally advantageous compared to continuous illumination as it does not increase the sample's temperature, which may have negative effects on cell viability.

To set high measures for the cytotoxicity tests, we expressed ChR2(D156A) in the human melanoma cell line BLM,^13^ as melanoma is renowned for its resistance apoptosis.^14^ To avoid cellular stress as a consequence of permanent ChR2(D156A) expression and potential activation by daylight, we generated a doxycycline-inducible transgenic TetOn-BLM cell line in the text referred to as the ChR2(D156A)-YFP BLM cell line.

### Relative calcium permeability of ChR2(D156A)

To test if the ChR2(D156A) Ca^2+^ permeability lies within the norm for ChR2 variants, that is, if ChR2(D156A) presents a good model for the study, we performed two-electrode voltage-clamp experiments on *Xenopus laevis* oocytes as previously described.^1^ To compensate for the small single channel conductance (~ 45 fS) and relatively low Ca^2+^ permeability intrinsic to ChR2s,^1, 15^ we raised extracellular Ca^2+^ to 80 mM. At negative holding potentials (*V*_*h*_ −120 mV), ChR2(D156A) activation triggered a large inward current with a biphasic rise time, characteristic for a fast light-activated Ca^2+^ entry into the cytosol and a secondary slower activation of the oocyte's endogenous Ca^2+^-sensitive chloride channels (CaCC).^1^ To qualitatively compare ChR2(D156A) Ca^2+^ transmittance to ChR2 wild-type and the most Ca^2+^-permeable variant CatCh,^1^ we rapidly removed cytosolic-free Ca^2+^ after light activation with the fast Ca^2+^-chelator BAPTA. BAPTA reduced the amplitudes of the secondary currents in ChR2(D156A)-expressing oocytes significantly more (85±5%) than in ChR2-expressing oocytes (66±7%, *P*<0.001), but less than in CatCh-expressing oocytes (96±2%, *P*<0.001). We therefore considered ChR2(D156A) a good representative for ChR2 variants.

### Chronic ChR2(D156A) activation kills human melanoma cells

**Figure 1:**
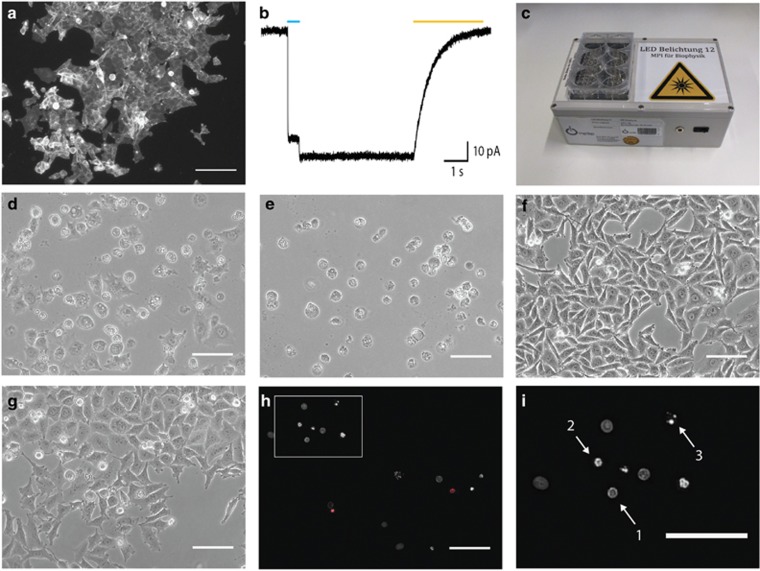
Illumination of ChR2(D156A)-YFP BLM cells induces morphological signs of cell death. (**a**) Fluorescence image of the ChR2(D156A)-YFP BLM cells 2 days after induction with 1 *μ*g/ml doxycycline. All cells show a bright, membrane-located fluorescence signal. (**b**) Whole-cell voltage-clamp recording showing a typical photocurrent evoked by a 500 ms long 473 nm (blue bar) light pulse. The channel is closed after 5 s with a 3 s long 593 nm (orange bar) light pulse. (**c**) Custom-designed battery-powered LED panel for *in vitro* experiments. (**d** and **e**) Continuous light-treatment of doxycycline-induced ChR2(D156A)-YFP BLM cells led to membrane blebbing and rounding up of cells after 2 days (**d**) and cell detachment after 3 days (**e**). Exposure of ChR2(D156A)-YFP BLM cells to light alone, without preceding doxycycline-induction (**f**, Ctrl Light) or inducing ChR2(D156A) expression without illumination (**g**, Ctrl Dox) for 3 days had no effect on cell viability. (**h** and **i**) Activating ChR2(D156A) for 2 days induced chromatin condensation and apoptosis. Late apoptotic cells are labeled by PI (**h**, red). Higher magnification of the boxed area (**i**) shows condensed chromatin forming a ring like structure (arrow 1), already fragmented chromatin forming a necklace-structure (arrow 2) and the final stadium of chromatin collapse (arrow 3). Hoechst33342 stained nuclei are shown in gray. Scale bars 100 *μ*m (50 *μ*m in I)

Forty-eight hours after addition of doxycycline to the cell culture medium, the ChR2(D156A)-YFP BLM cell line showed strong membrane-located YFP fluorescence (Figure 1a). Whole-cell patch-clamp recordings from ChR2(D156A)-YFP BLM cells confirmed a typical, long-lasting light-evoked (473 nm) photocurrent with a current density of 1.4±0.9 pA/pF (*N*=4, mean±S.D., Figure 1b) that could be terminated by illumination with a second, red-shifted wavelength (593 nm).^2^ The prolonged ChR2(D156A) current activation upon a brief blue light flash enabled us to use a pulsed illumination protocol (1 s light on, 10 s light off) for our subsequent cytotoxicity studies, minimizing secondary light and heat effects.

For chronic pulsed illumination of ChR2(D156A)-YFP BLM cells in the cell culture incubator, we designed a battery-powered LED panel carrying a tray for six-well cell culture plates (Figure 1c). Exposing doxycycline-induced ChR2(D156A)-YFP BLM cells for 48 h to pulsed light induced clear morphological changes and reduced cell viability consistent with apoptosis, that is, membrane blebbing and the appearance of a rounded morphology (Figure 1d) that eventually led to death and complete cell detachment after 3 days (Figure 1e). In contrast, chronic blue light illumination over 3 days in control cells (Ctrl Light) that had not received preceding doxycycline treatment and therefore did not express ChR2(D156A)-YFP showed no changes in cell viability (Figure 1f). This was rather surprising as it disproves the expected phototoxicity of prolonged high-intensity blue light illumination. Equally, ChR2(D156A)-YFP expression by doxycycline induction of ChR2(D156A)-YFP BLM cells without subsequent illumination (Ctrl Dox) had no effect on cell morphology (Figure 1g). In summary, the prolonged depolarization combined with an increasing intracellular Ca^2+^ load may pose concerns in light of a chronic treatment using ChR2s.

To visualize cell death and track changes in chromatin morphology indicative of apoptosis, we stained the nuclei with Hoechst and propidium iodide (PI),^16^ latter intercalating into the late apoptotic and necrotic cells. Treated (doxycycline-induced and illuminated) ChR2(D156A)-YFP BLM cells clearly showed different apoptosis-associated stages of chromatin condensation and fragmentation over the 3-day illumination period (Figures 1h and i), indicative of efficient optogenetic induction of controlled cell death, which were not apparent in Ctrl Light and Ctrl Dox cells.

**Figure 2:**
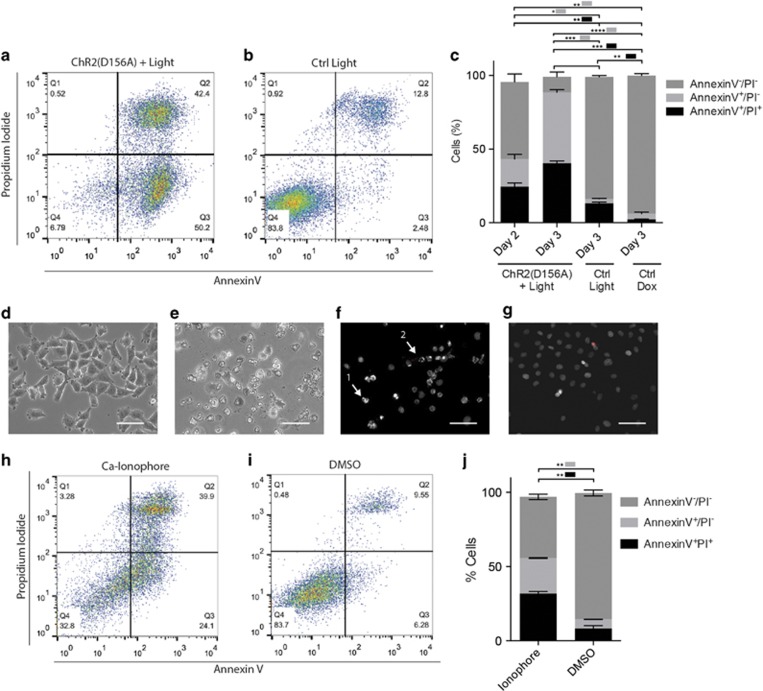
Ca^2+^ ionophore and ChR2(D156A) optogenetic light treatment have similar effects on BLM cell viability. (**a**-**c**) Flow cytometric quantification of apoptosis using Annexin-V and propidium iodide (PI). Representative plots after 3 days of treatment with ChR2(D156A)+light (**a**) or light only (Ctrl Light, **b**). Q3; early apoptotic population, Q2; late apoptotic population. (**c**) Quantification, mean±S.D. from three independent experiments. (**d**-**g**) Changes in cell (**d**, **e**) and chromatin morphology (**f**,**g**) after 24-h treatment with DMSO and 5 *μ*M Ca^2+^ ionophore A23187. Ionophore treatment led to cell detachment, membrane blebbing (**f**; arrow 1) and chromatin condensation (**f**; arrow 2). Late apoptotic cells are co-labeled by PI (red), Hoechst33342 stained nuclei are shown in gray, scale bars 100 *μ*m. (**h**-**j**) Flow cytometry analysis of apoptotic cells using Annexin-V/PI. Representative plots after 24 h of treatment with (**h**) 5 *μ*M ionophore A23187 and (**i**) DMSO, (**j**) Quantification, mean±S.D. from two independent experiments. *P<0.05, **P<0.01, ***P<0.001 and ****P<0.0001

The percentage of apoptotic cells after 2 and 3 days of treatment was quantified with flow cytometry using Annexin V/PI staining. One of the earlier events of apoptosis includes transfer of phosphatidylserine, which ordinarily resides on the cytoplasmic surface of the membrane, to the outer cell surface. Annexin V has high affinity for phosphatidylserine and labels apoptotic cells. The Annexin V/PI assay clearly showed more early (Annexin V^+^/PI^−^) and late apoptotic (Annexin V^+^/PI^+^) cells in the treated ChR2(D156A)-YFP BLM group (Figure 2a) compared to the Ctrl Light (Figure 2b) and Ctrl Dox groups (Figure 2c). The bar graph in Figure 2c quantifies the results, confirming a significantly higher percentage of early and late apoptotic cells in the treated ChR2(D156A)-YFP group after 2 days (early apoptotic: 18.7±3.2% (*P*<0.01 *versus* Day 3 Ctrl Dox, *P*<0.05 *versus* Day 3 Ctrl Light); late apoptotic: 24.6±2.4% (*P*<0.01 *versus* Day 3 Ctrl Dox and Ctrl Light) and 3 days (early apoptotic: 48.2±1.8% (*P*<0.0001 *versus* Day 3 Ctrl Dox, *P*<0.001 *versus* Day 3 Ctrl Light; late apoptotic: 40.3±1.7% (*P*<0.001 *versus* Day 3 Ctrl Dox and Ctrl Light) than in the 3 days Ctrl Light (early apoptotic: 2.96±0.42% late apoptotic: 13.2±0.87%) and Ctrl Dox (early apoptotic: 3.76±1.18% late apoptotic: 2.33±0.21%) groups. However, the Ctrl Light group showed also a small but significant increase in late apoptotic cells compared to the Ctrl Dox group (*P*<0.01), indicating some phototoxicity of the light treatment alone.

### Is Ca^2+^ involved in melanoma cell death?

To test if BLM melanoma cells were susceptible to apoptosis induction through sustained depolarization combined with a rise in cytosolic Ca^2+^ we compared side-by-side the effects of the Ca^2+^ ionophore A23187 and light-activation of ChR2(D156A) on cultured BLM melanoma cells.

Twenty-four hours after A23187 treatment we clearly observed morphological signs of apoptosis in BLM cells similar to those observed under optogenetic treatment, such as membrane blebbing, chromatin condensation and cell detachment (Figures 2d and g). Also, the Annexin V/PI flow cytometry assay after ionophore treatment gave similar results to those observed after optogenetic treatment: a significant increase of early (Annexin V^+^/PI^−^: 23.9±0.3%, *P*<0.01) and late (Annexin V^+^/PI^+^: 41.2±1.8%, *P*<0.01) apoptotic cells compared to the DMSO control group (early apoptotic: 6.2±0.1% late apoptotic: 8.2±1.9% Figures 2h and j).

**Figure 3:**
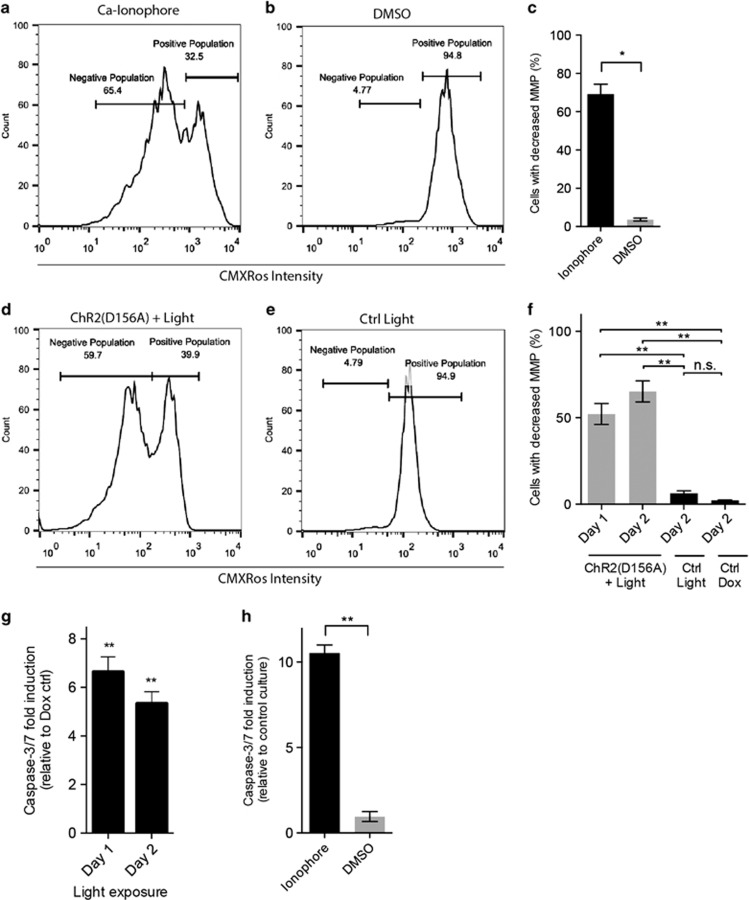
Side-by-side comparison of the apoptotic pathways induced by ChR2(D156A) and the Ca^2+^ ionophore A23187, respectively. (**a**-**f**) Flow cytometric analysis of MMP using CMXRos. Representative plots after 24 h of treatment with (**a**) 5 *μ*M A23187 and (**b**) DMSO, (**c**) Quantification. Representative plots after 2 days of treatment with ChR2(D156A)+light (**d**) or light only (Ctrl light, **e**), (**f**) Quantification. (**g** and **h**) Induction of executor caspase 3 determined by the Ac-DEVD-AMC cleavage assay, (**g**) optogenetic induction relative to Ctrl Dox after 1 and 2 days of light activation, (**h**) ionophore and DMSO induction after 24 h relative to non-treated control cultures. Mean±S.D. from two independent experiments in (**c**) and (**h**) and from three independent experiments in (**f**) and (**g**). *P<0.05, **P<0.01. n.s., non-significant

A clear indicator of mitochondrial overstrain and mitochondrial death pathway induction are the loss of the mitochondrial transmembrane potential (MMP), which can be visualized on a flow cytometer by the mitochondrial dye CMXRos, accumulating in healthy mitochondria. Twenty-four hours after ionophore treatment, a significantly higher percentage of mitochondria showed a decreased MMP (negative cell population, 69.1±5.2% *P*<0.05) compared to DMSO controls (4.25±3.1%), indicative of mitochondrial Ca^2+^ overstrain (Figures 3a and c). Analogously, 2 days of ChR2(D156A) light treatment resulted in a similarly significant decrease in MMP (65.3±6.1%) compared to the Ctrl Light (6.3±1.4% *P*<0.01) and Ctrl Dox groups (2.3±0.2% *P*<0.01, Figures 3d and f). Loss of the MMP causes the release of cytochrome c from the mitochondria, formation of the apoptosome and activation of caspase-3 (Figure 5).^17^ We therefore determined the levels of active caspase-3 in lysates of treated ChR2(D156A)-YFP BLM cells and ionophore-treated BLM cells. Already after 1 day of optogenetic treatment we found a significant, 6.6±0.6-fold upregulation of active caspase-3 relative to the Ctrl Dox group (*P*<0.01, Figure 3g). Again, similar results were observed 24 h after ionophore treatment with 10.6±0.5-fold elevated caspase-3 levels compared to the DMSO controls (*P*<0.01; Figure 3h).

**Figure 4:**
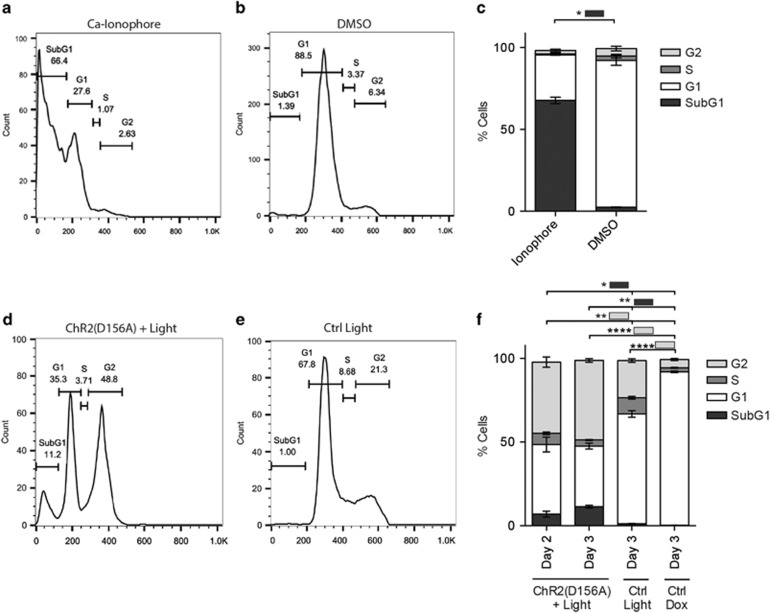
Sub-G1 cell cycle assays of ionophore and optogenetic treated BLM cells. (**a**-**c**) Representative plots after 24 h of treatment with (**a**) 5 *μ*M A23187 or (**b**) DMSO, (**c**) Quantification, mean±S.D. from two independent experiments. (**d**-**f**) Representative plots after three days of treatment with ChR2(D156A)+light (**d**) or light only (Ctrl light, **e**), (**f**) Quantification, mean±S.D. from three independent experiments. *P<0.05, **P<0.01 and ****P<0.0001

Terminally, apoptosis leads to DNA fragmentation indicative of cell death. DNA fragmentation was analyzed with the sub-G1 flow cytometry assay. Twenty-four hours of ionophore treatment led to the appearance of a significant hypodiploid, apoptotic sub-G1 cell population (67.8±1.9%, Figure 4a) compared to the DMSO control (2.5±0.2%, *P*<0.05; Figures 4b and c). Similarly, ChR2(D156A) light treatment significantly increased the amount of cells in the sub-G1 peak compared to the controls (6.9±1.7% after 2 days, *P*<0.05 *versus* Day 3 Ctrl Light and Ctrl Dox; 11.3±0.9% after 3 days, *P*<0.01 *versus* Day 3 Ctrl Light and Ctrl Dox; Figures 4d and f). Additionally, we observed a G2/M cell cycle arrest in ChR2(D156A)-YFP BLM cells after 2 days of light treatment, with 42.6±3.1% of cells (*P*<0.01 *versus* Day 3 Ctrl Dox and Ctrl Light) arrested in the G2-phase, which increased to 47.6±1.1% (*P*<0.0001 *versus* Day 3 Ctrl Dox and Ctrl Light) over the 3-day illumination period (Figure 4f). A G2/M arrest followed by selective tumor cell apoptosis has previously been reported for pharmacological treatment of breast and prostate cancers.^18^ Also here the Ctrl Light group showed a significant increase in G2/M cell cycle arrest compared to the Ctrl Dox (*P*<0.0001). Although the effect was significantly smaller than in the treated group, some phototoxicity seems to be mediated by light treatment alone. We explain the lack to detect a G2/M arrest in ionophore-treated BLM cells by the fact that apoptosis is initiated much faster so that at the time point of the assay most cells had already died.

**Figure 5:**
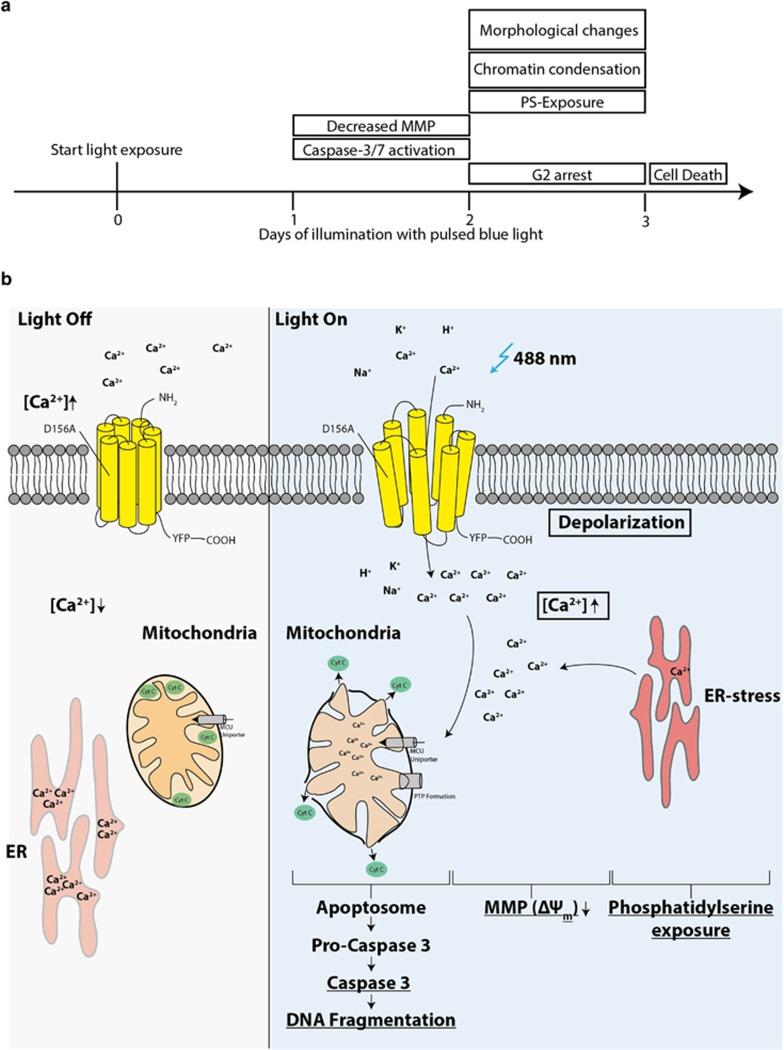
Hypothetical apoptotic pathway upon light-activation of ChR2(D156A) in BLM melanoma cells. (**a**) Schematic time-line of apoptotic events in treated ChR2(D156A)-YFP BLM cells. (**b**) Proposed apoptotic pathway and hallmarks of optogenetically induced cell death. Blue light activation of ChR2(D156A) leads to cell depolarization and potentially to an increase of cytosolic Ca^2+^, leading to Ca^2+^ uptake into the mitochondria and the endoplasmatic reticulum (ER). Depolarization may potentiate cytosolic free Ca^2+^ through ER stress, leading to Ca^2+^ release from the ER. The sharp rise of cytosolic Ca^2+^ activates the mitochondrial uniporter MCU, which increases Ca^2+^ in the mitochondrial matrix, a stimulus for the induction of the permeability transition (PTP formation) resulting in a decreased mitochondrial membrane potential (MMP) and the release of cytochrome c (Cyt c). Cyt c activates the ‘apoptosis executor' caspase 3 via the formation of a complex known as the apoptosome, terminally leading to DNA fragmentation. Phosphatidylserine is switched to the outer leaflet of the plasma membrane during apoptosis acting as an ‘eat-me' signal for adjacent phagocytes

The above comparative results show that BLM cells are susceptible to prolonged depolarization combined with a rise in cytosolic Ca^2+^ and that optogenetic treatment induces the mitochondrial death pathway. They also suggest that the small phototoxic effects of the light treatment alone induce apoptosis independent of mitochondria, as there were no differences in MMP, active caspase-3 levels or sub-G1 cell populations detected between the Ctrl Light and Ctrl Dox groups. The difference between ionophore and optogenetic treatment lies in the timeline: while ionophore-induced apoptosis is already completed after 24 h, optogenetically mediated apoptosis required 3 days (Figure 5a). The difference can be explained by the bulk Ca^2+^-influx over the plasma membrane and thus large depolarization mediated by the ionophore, which contrasts the small single channel conductance of ChR2(D156A).^2, 15^

Figure 5b summarizes the proposed pathway for optogenetic apoptosis induction in ChR2(D156A)-YFP BLM cells. In summary, our results are in good agreement with previous studies showing that both, depolarization and a rise in cytosolic Ca^2+^ play a pro-apoptotic role in human melanoma cells, upregulating in parallel the mitochondrial death pathway.^19, 20, 21^

### Anti-tumor efficacy of ChR2(D156A) *in vivo* in a xenograft mouse model

**Figure 6:**
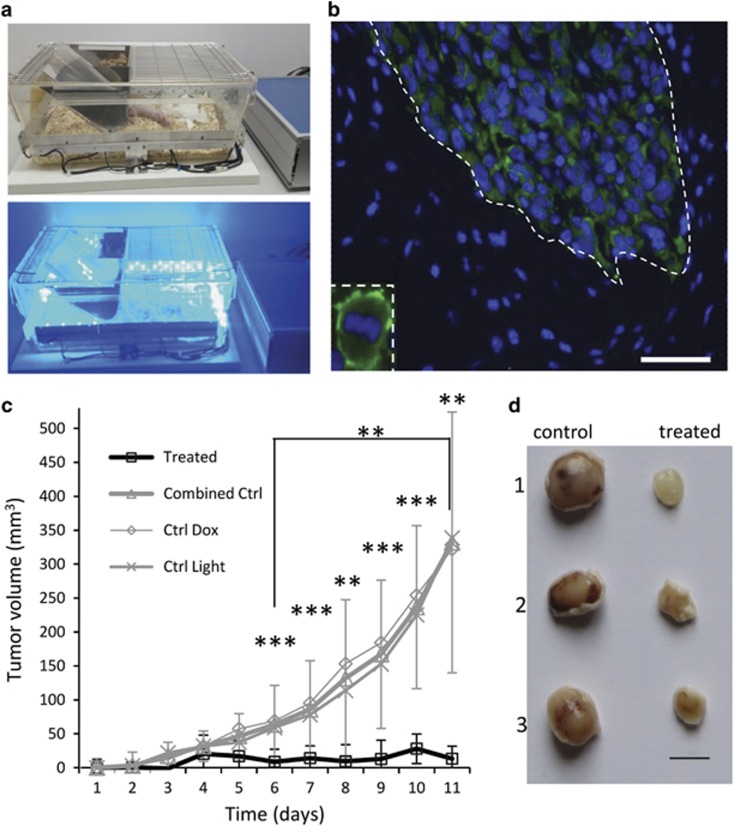
Light stimulation inhibited growth of subcutaneous melanoma xenografts in nude mice. (**a**) LED-illumination cages used for xenograft simulation. (**b**) *In vivo* expression of ChR2(D156A)-YFP after doxycycline induction visualized by green YFP fluorescence. The tumor is clearly delineated and the membrane location of ChR2(D156A) is evidently visible in the enlarged insert. Scale bar 50 *μ*m. (**c**) Growth curve of the tumors after doxycycline induction and/or illumination indicated as changes in extrapolated tumor volume. All experimental mice were injected with inducible BLM cells and the experiment was started when the tumors reached a diameter of 5 mm (here set to 0), by either inducing ChR2(D156A)-YFP expression with doxycycline (Ctrl Dox, *N*=5) or by illumination of the tumor (Ctrl Light, *N*=6) or by both (treated, *N*=11). Ctrl Dox and Ctrl Light, since almost identical, were averaged to the ‘combined Ctrl'. (**d**) Examples of excised tumors on day 11 of the treatment. Left lane: (1) and (2) Ctrl Light, (3) Ctrl Dox, right lane: examples of treated tumors. Scale bar 5 mm. **P<0.01, ***P<0.001

Such effective killing of melanoma cells by optogenetic treatment prompted us to test the effects *in vivo*. We transplanted ChR2(D156A)-YFP BLM cells into the subcutaneous spaces of both lower flanks of immune-compromised nude mice. The transplanted mice were then randomly allocated to one of three treatment groups: the ‘treated group' received doxycycline and light treatment (*N*=11), the Ctrl Dox group received only doxycycline treatment (*N*=5) and the Ctrl Light group received only light treatment (*N*=6). When one of the tumors in either flank reached ~5 mm in diameter, the 11-day treatment regime was immediately and individually started to guarantee a similar efficacy of illumination, as the light intensity is attenuated by tumor depth.^22, 23^ ChR2(D156A) expression was induced in tumors of the ‘treated' and Ctrl Dox groups by replacing food pellets with doxycycline (200 mg/kg) containing pellets. Pulsed (1 s on - 10 s off) blue light illumination for 12 h per day over the 11-day treatment period was achieved by placing the cages of the ‘treated' and Ctrl Light groups into a custom-made LED-lining (Figure 6a). Mice were euthanized at day 11 of the treatment, tumors collected and pathologically examined as described below. Tumors of mice that were fed doxycycline consisted exclusively of yellow fluorescing cells, indicative of ChR2(D156A)-YFP expression (Figure 6b). While tumors displayed rapid and continued growth in both control groups during the course of treatment, tumor growth halted in the optogenetically treated group and in some cases tumor size even decreased (Figures 6c and d). The mean tumor volume after 11 days of treatment was 368±71 mm^3^ in the Ctrl Dox, 380±40 mm^3^ in the Ctrl Light and 37±9 mm^3^ in the treated group (mean±S.E.M.), significantly smaller than that of both control groups (*P*<0.01, Figure 6c). As the growth curves of the Ctrl Dox and Ctrl Light groups were virtually identical, we combined the two control groups in the subsequent pathological analyses.

**Figure 7:**
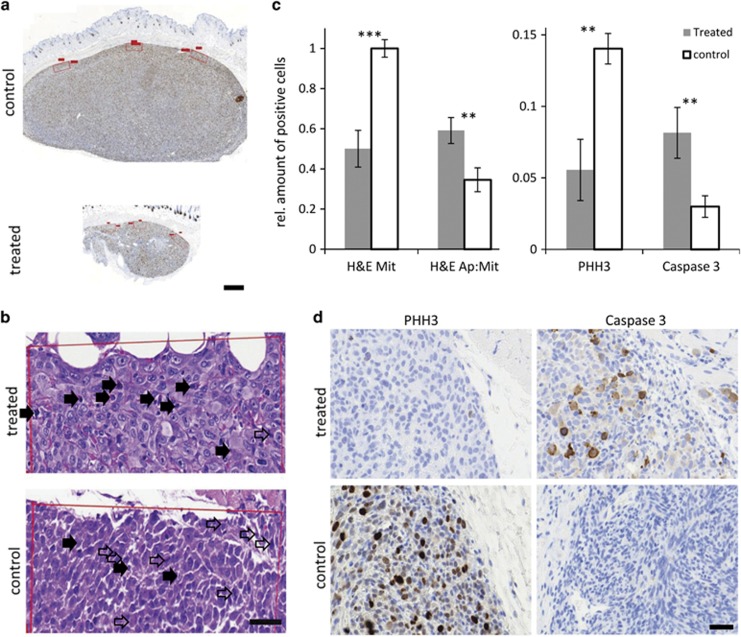
Immunohistochemistry on paraffin sections of excised xenograft tumors. (**a**) Paraffin sections of a control (top) and a treated (bottom) tumor showing the smaller *z*-axis extension of the treated tumor and the locations of the annotation fields for scoring. Scale bar 500 *μ*m. (**b**) Example H&E-stained sections (most superficial 100 *μ*m of tumor tissue) of treated (top) and control (bottom) tissue, showing the clearly increased apoptosis-to-mitosis ratio in treated tumors. Filled arrows mark apoptoses, open arrows indicate mitoses. (**c**) Quantification of scoring. Mean±S.E.M. (**d**) Examples from immunohistochemically stained control and treated subcutaneous xenograft tissue. Scale bars (**c** and **d**) 50 *μ*m. **P<0.01, ***P<0.001

Paraffin-embedded tumors were sectioned, which confirmed that treated tumors were also in their *z*-axis extension much smaller than control tumors after 11 days of treatment (Figure 7a). For each section, three representative annotation fields located centrally and peripherally in the tumor with a width of 300–500 *μ*m and a maximum depth of 200 *μ*m were scored by two independent observers to whom the identity of sections was unknown (Figure 7a). To determine the amount of mitotic and apoptotic nuclei, sections were stained with hematoxylin and eosin (H&E) and positive cells counted by trained pathologists (Figure 7b). As the standard determination of mitosis per high power field is not a true index of mitosis and varies in tumors due to area differences in cellularity, we normalized for cellularity by calculating the ratio of mitotic cells per total cell number in each annotation field before averaging. As expected, non-treated tissue contained significantly more mitotic cells (4.8±0.3%, mean±s.e.m.) than treated tumor tissue (2.4±0.4%, mean±s.e.m., *P*<0.001, Figure 7c). As an estimate of tumor growth dynamics, we then determined the apoptosis-to-mitosis ratio,^24^ which was again significantly elevated in treated tissue (0.59±0.05, mean±s.e.m.) compared to controls (0.34±0.06, mean±s.e.m., *P*<0.01), explaining the cessation of tumor growth under optogenetic treatment (Figure 7c). To relate the pro-apoptotic and anti-proliferative effects of the *in vivo* treatment to our *in vitro* data, we performed PHH3 (phosphohistone H3)^25^ and cleaved caspase-3 immunohistochemistry on additional paraffin sections. PHH3 is considered an effective marker to visualize the mitotic index and differentiate between mitotic figures and apoptotic bodies, as phosphorylation at histone H3 almost exclusively occurs during mitosis^26^ and is absent during apoptosis.^27^ As expected, PHH3 labeling paralleled the mitotic count, which was significantly increased in non-treated tissue (14±2.2%) compared to treated tissue (5.6±1.5%, *P*<0.01, Figures 7c and d). In line with our *in vitro* data, also caspase-3 was significantly elevated in treated tissue (8.1±1.0%, mean±s.e.m.) compared to control tissue (3.0±1.0%, mean±s.e.m., *P*<0.01, Figures 7c and d).

Our *in vivo* results indicate the potential value of a ChR2-based optogenetic cancer therapy. As illumination of the xenograft tissue was restricted to daytime in order to comply with animal ethics, the effects of chronic illumination would expectedly be enhanced.

## Discussion

The majority of recent translational studies mention the potential phototoxicity of prolonged ChR2 activation in a clinical setting,^3, 28^ as ChR2s require above human safety threshold radiation intensities for activation.^7^ As such cytotoxic effects have never been quantified, we here mended this gap and chronically illuminated ChR2(D156A)-expressing BLM melanoma cells. We could clearly show that 3 days of pulsed light killed all melanoma cells. The cytotoxic effects are expected to be enhanced in neurons, as cancer cells are almost resistant to killing. However, at least under the given illumination conditions, cell death was not primarily mediated by the suspected blue light phototoxicity but rather by prolonged membrane depolarization, which had previously been demonstrated to potentiate apoptosis induction in melanoma cells.^19^ Nonetheless, a slight but significant increase in the amount of apoptotic cells was also observed in cell culture experiments in the Ctrl Light group (see Figures 2c and 4f) that may be further enhanced when wild-type ChR2 is employed, which requires sustained light activation due to its fast off-kinetics and 100-times higher light intensities. However, differences in the Ctrl Light and Ctrl Dox groups were absent in the *in vivo* xenograft experiments, suggesting that phototoxicity is low. The similarity of the sequences of apoptotic events triggered optogenetically and by the calcium ionophore A23187 confirmed activation of the mitochondrial death pathway under optogenetic treatment. Directly light-induced apoptosis on the other hand seems to be independent of the mitochondrial pathway, as no significant changes in MMP, active caspase-3 levels or a sub-G1 population were detected in the Ctrl Light group (see Figures 3f and 4e). Illumination regime, light intensity and Ca2+ transmittance of the used ChR2 variant all contribute to the final cytotoxic effect, which is difficult to foresee and to experimentally determine, as long-term high-intensity illumination leads to heat production and phototoxicity and Ca^2+^ *per se* is a finely tuned second messenger with diverse roles. Nonetheless, ChR2 variants that had been developed for improved clinical applicability with increased light response amplitudes and slowed kinetics will increase the overall cell depolarization and Ca^2+^ transmittance, which renders them potentially more toxic.^1, 2^ Recently developed Opto-GPCRs that are coupled to native cellular pathways^3, 9, 29, 30^ and are 1000-fold more light sensitive may therefore present alternative safer tools for the treatment of neurological disorders in human patients.

Skin cancer is a devastating disease with rapidly increasing incidence.^31^ Melanoma treatment remains particularly challenging due to the disease's frequent relapse mediated by the cancer cells' acquired resistance to treatment^10, 11, 12, 32^ in terms of changes in specific apoptosis signaling.^12^ Although relying on apoptosis, optogenetic treatment presents a highly ubiquitous apoptotic stimulus that melanoma cells appear to be unable to evade by specific pathway accommodations.

We demonstrate in both, melanoma cell culture and xenografts that melanoma cell viability decreases under optogenetic light treatment by activating the mitochondrial death pathway. Expression of ChR2s in cancer cells may thus be worthwhile to be evaluated as a potential cancer therapeutic, alone or in combination with existing treatments to boost their efficacies. A combination with photoactivatable agents such as light-activated antibodies that harness the immune system^33, 34^ could be attractive as local photoactivation adds treatment specificity and spares healthy tissue. Precise light delivery methods have been developed for photodynamic cancer therapy, and more recently, for optogenetics.^22^ A second level of treatment specificity could be given by selective expression of ChR2 in cancer cells. Molecular tools for genetically targeted expression are under active investigations with some promising genetic tools arising, such as cancer cell-specific promoters^35, 36^ and systemically deliverable viruses that efficiently target cancer cell surface proteins.^37, 38, 39^ The fact that optogenetics induces controlled apoptotic cell death and not necrosis reduces the risk of a spreading inflammation adding a third level of specificity.

In conclusion, the current study demonstrates another opportunity for the optogenetic toolbox to extend beyond excitable cells to inhibit cancer cell proliferation.

## Materials and Methods

### BLM human melanoma cell line

The highly invasive human melanoma cell line BLM, a subline of the aggressive human melanoma cell line BRO derived from a biopsy of a malignant human primary melanoma of the skin,^13^ was obtained from H. Büning (Center for Molecular Medicine, University of Cologne, Germany). BLM cells were maintained in 1640 RPMI medium (Sigma-Aldrich, St. Louis, MO, USA) supplemented with 10% FCS (Biochrom, Berlin, Germany), 100 Units/ml Penicillin (Sigma-Aldrich), 100 mg/ml Streptomycin (Sigma-Aldrich), 1% Glutamin (Sigma-Aldrich) and 1% non-essential amino acids (Gibco, Carlsbad, CA, USA) at 37 °C and 5% CO_2_. The cells were passaged every 3-4 days at a 1:10 ratio using trypsin/EDTA.

#### Inducing cell death with ChR2(D156A) and light in pTRE3G-ChR2(D156A)-YFP BLM cells

On the first day, pTRE3G-ChR2(D156A)-YFP BLM cells were seeded in six-well plates (TPP) at 8 × 10^4^ cells/well in doxycycline-free BLM medium. ChR2(D156A)-YFP expression was induced for 2 days with 1 *μ*g/ml doxycycline (Pfizer, New York, NY, USA), following replacement of medium with doxycycline-free BLM medium containing 1 *μ*M all-trans retinal. Immediately afterwards, cells were put on the LED light exposure chamber (see below) at 37 °C and 5% CO_2_.Doxycycline and all-trans retinal were replenished every 24 h.

#### Inducing cell death with the calcium ionophore A23187 in pTRE3G-ChR2(D156A)-YFP BLM cells

pTRE3G-ChR2(D156A)-YFP BLM cells were seeded in six-well plates (TPP) at 8 × 10^4^ cells/well in doxycycline-free BLM medium and incubated at 37 °C and 5% CO_2_ for two days. Afterwards, the medium was replaced with doxycycline-free BLM medium containing 5 *μ*M calcium ionophore A23187 (Sigma-Aldrich).

### Molecular biology

#### pTRE3G-ChR2(D156A)-YFP plasmid construction

The humanized ChR2(D156A)-YFP gene (*hChR2(D156A)-YFP*) was cloned into the pTRE3G-IRES plasmid (Clontech, Mountain View, CA, USA) by using the In-Fusion HD Cloning Kit (Clontech). The pTRE3G-IRES plasmid was digested with *EagI* and *MluI* and the PCR-amplified *hChR2(D156A)-YFP* (F: TCTATCGATCGGCCGGATCCACCATGGACTACG, R: TAGCCATATGACGCGTCTTTACTTGTACA GCTCGTCC) inserted.

#### Generation of inducible pTRE3G-ChR2(D156A)-YFP BLM cell line

All transfections were performed with the Xfect TM transfection kit (Clontech) according to the manufacturer's protocol. First, the wild-type BLM cells were transfected with the pEF1a-Tet3G plasmid (Tet-On 3G Expression System, Clontech) and grown under geneticin (1500 *μ*g/ml) selection for 2 weeks. Single cells were sorted with a FACS Aria III flow cytometer (Becton Dickinson, NJ, USA), expanded in geneticin selection medium and resistant clones individually probed for Tet-ON 3G transactivator expression levels by a luciferase assay with the inducible plasmid pTRE3g-Luc (Tet-On 3G Expression System, Clontech) according to the manufacturer's protocol. The highest expressing clone (Tet-on 3G BLM) was subsequently co-transfected with the pTRE3G-ChR2(D156A)-YFP plasmid and a linear puromycin selection marker and grown under puromycin (0.75 *μ*g/ml) selection for 2 weeks. Twenty-four hours before single cell sorting, 1 *μ*g/ml doxycycline was added to the culture medium in order to induce ChR2(D156A)-YFP expression. Single cells with high YFP signal were sorted in 96-well plates using the FACS Aria III (Becton Dickinson) and the clone with highest ChR2(D156A)-YFP expression levels qualitatively selected for further experiments (in this manuscript referred to as ChR2(D156A)-YFP BLM cell line).

### Electrophysiology

#### Two-electrode voltage clamp in Xenopus oocytes

The genes encoding wild-type ChR2,^40^ ChR2(D156A)^2^ and CatCh^1^ were inserted into the pGEM-HE vector and expressed in oocytes of *X. laevis* as described previously.^1^ Oocytes were obtained by collagenase treatment after partial ovarectomy and injected with 30 ng of *in vitro*-transcribed mRNA (SP6 mMessge mMachine kit, Ambion, Foster City, CA, USA). After mRNA injection, oocytes were incubated in all-*trans* retinal (1 *μ*M) and were kept in ORI buffer (90 mM NaCl, 2 mM KCl, 2 mM CaCl_2_ and 5 mM MOPS, pH 7.4) containing 1 mg/ml gentamycin at 18 °C for 2–4 days. In subsequent two-electrode voltage-clamp experiments, oocyte Ringer contained 80 mM Ca^2+^ and light activation was achieved with a 75-W xenon arc lamp and a 450±25 nm band filter, the light of which was coupled into a 1-mm light guide with an output of ~5 mW/mm^−5^. To suppress calcium-activated chloride channel (CaCC) currents, we injected 50 nl of a 20 mM solution of the fast Ca^2+^-chelator BAPTA into each oocyte (~1 mM final concentration in the oocyte). The relative decrease in current amplitude upon BAPTA treatment was taken as a measure for the qualitative Ca^2+^ permeability.

#### Whole-cell patch-clamp recordings in pTRE3G-ChR2(D156A)-YFP BLM cells

Recordings were performed on an inverted Zeiss Axiovert 35M microscope. Recordings were made in the whole-cell configuration using borosilicate glass pipettes (Harvard Apparatus GC150F-10) pulled with a Zeitz DMZ-Universal puller ranging from 2 to 6 MΩ. The pipette solution contained (in mM): 123 K-Gluconate, 7 KCl, 1 MgCl_2_, 5 ATP_Na2_, 10 EGTA, 10 HEPES (pH 7.4, KOH). The bath solution contained (in mM): 135 NaCl, 4 KCl, 1 MgCl_2_, 2 CaCl_2_, 10 glucose, 10 HEPES (pH 7.4, NaOH). Signals were amplified with an Axopatch 200B Amplifiter, low pass filtered at 5 kHz and digitized at 50 kHz with an Axon Digidata 1440A. Acquisition and analysis was performed using pClamp software (Molecular Devices, Biberach, Germany). Membrane voltage was clamped at −60 mW and light pulses were supplied by two solid state lasers (Pusch Opto Tech GmbH, Wettenberg, Germany; *λ*=473 nm, *λ*2=593.5 nm), which were coupled to a 400 *μ*m optic fiber. Light pulses were applied by a fast computer-controlled shutter (Uniblitz LS6ZM2, Vincent Associates, Rochester, NY, USA). YFP signals were observed using 473 nm excitation through a GFP filter set (#31001, Chroma Technology, Bellows Falls, VT, USA).

### *In vitro* apoptosis assays

#### Hoechst33342 chromatin staining

Cells were labeled directly in the six-well plate with 50 *μ*g/ml Hoechst33342 (Sigma-Aldrich) for 5 min at 37 °C and washed with PBS. PI was added in the volume 5 *μ*g/ml immediately before visualization with an inverted Zeiss Axiovert 100 Microscope.

#### Annexin V/propidium iodide flow cytometry assay

Cells were washed with PBS, dissociated with 200 *μ*l trypsin/EDTA and centrifuged for 6 min at 1000 × *g*. The pellet was resuspended in 100 *μ*l Annexin V-APC binding buffer 100 mM HEPES, 140 mM NaCl, 25 mM CaCl2 and pH 7.4 in water. One microliter Annexin V-APC staining solution (Biolegend, San Diego, CA, USA) was added and the cells incubated for 15 min at room temperature in the dark. Immediately before measuring with the LSRII SORP flow cytometer (Becton Dickinson), 400 *μ*l Annexin V binding buffer and 5 *μ*l PI solution (1 mg/ml in PBS, Sigma-Aldrich) were added. The Annexin V-APC and PI signals were collected on a logarithmic scale using a 640 nm laser in combination with a 660 nm filter (20 nm bandwidth) and a 488 nm laser in combination with a 695 nm filter (40 nm bandwidth), respectively. Analysis was performed with FlowJo software (Version 10.0.7, Treestar Inc. Ashland, OR, USA).

#### Mitochondrial membrane potential

Cells were washed with PBS, dissociated with 200 *μ*l trypsin/EDTA and centrifuged for 6 min at 1000 × *g*. The pellet was resuspended with 300 *μ*l RPMI-1640 (Sigma-Aldrich) containing 100 nM MitoTracker red CMXRos (Invitrogen, Carlsbad, CA, USA) and incubated at 37 °C for 1 h. Cells were subsequently centrifuged for 6 min at 1000 × *g*, resuspended in 300 *μ*l PBS and analyzed with the LSRII SORP flow cytometer (Becton Dickinson). The CMXRos signal was collected on a logarithmic scale with a 561 nm laser in combination with a 610 nm filter (20nm bandwidth) and data analyzed using FlowJo software.

#### Caspase-3/7 activity

Cells were washed with PBS, dissociated with 200 *μ*l trypsin/EDTA and centrifuged for 6 min at 1000 × *g*. Harvested cells were resuspended with 50 *μ*l lysis buffer (0.1% Triton X-100 in PBS, Sigma-Aldrich), incubated on ice for 10 min and centrifuged for 5 min at 16 000 × *g* at 4 °C. The supernatant was carefully transferred into a 96-well plate (Greiner Bio-One, Frickenhausen, Germany) containing 150 *μ*l HEPES (40 mM HEPES pH 7.5, 20% Glycerol, 5 mM Dithiothreitol) and 50 *μ*M caspase- 3/7 substrate (Ac-DEVD-AMC). The reactions were incubated for 30 min at 37 °C in the dark before caspase activity was measured on a multimode reader (Infinite Pro 200, Tecan; ex: 368/9 nm, em: 475/20 nm). The caspase-3/7 fold induction was determined as the activity ratio of treated *versus* non-treated sample.

### *In vivo* xenograft model

#### Establishment in Swiss nude mice

Experiments were performed on immune-deficient Swiss Nude (Crl:NU(Ico)-*Foxn1*^*nu*^, Charles River) mice maintained in a quarantine cupboard equipes with isolated filtered ventilation under a standard 12-h light-dark cycle. All animal experiments were performed in accordance with the Swiss Federal Animal Protection Act and approved by the animal research committee of Bern (approval number BE5-14).

The xenograft tumor model was established by subcutaneous injections of 2 × 10^6^ doxycycline-inducible pTRE3G-ChR2(D156A)-YFP BLM cells 100 *μ*l of DMEM culture medium into both flanks of 4-week-old Swiss Nude mice under Isofluorane anaesthesia with a 18G needle. Care was taken to create a single bubble of cells beneath the skin and to avoid spread of the cells. The injection sites were capped with liquid plaster (OPSITE spray) to avoid infection. The tumor size was measured on a daily basis using a calliper. When one of the tumors reached a diameter of ~5 mm, the mice were divided randomly into three treatment groups, (1) receiving doxycycline and light treatment (treated group), (2) receiving only doxycycline treatment (Ctrl Dox) and (3) receiving only light treatment (Ctrl Light). For the treated and Ctrl Dox groups, food pellets were replaced by pellets containing 200 mg/kg doxycycline (TD.00502, Teklad 2018 Global Rodent diet, Harlan Laboratories, Venray, the Netherlands) in order to induce ChR2(D156A) expression. Concomitantly, the treated and Ctrl Light groups were moved into LED-lined cages (see Figure 6a and exposed to pulsed (1 s on, 10 s off) blue light for 11 days. During treatment, tumor size was continuously measured on a daily basis and tumor volume extrapolated by using the formula *V* (mm^3^)=length × width^2^ × 1/2, where *L* and *W* represent the largest and the smallest diameters, respectively.

#### Immunohistochemistry on excised tumors

Excised tumors were fixed in formalin for 24 h and embedded in paraffin. Subsequent processing was performed double-blind: the tissue blocks were randomly numbered for sectioning and immunohistochemistry and analysis was performed by observers blinded to treatment. The tumor containing paraffin-blocks were randomly numbered (11 treated, 11 untreated mice) and sectioned at 2.5 *μ*m by a blinded histologist. Sections were stained with H&E and antibodies against Ki-67 (rabbit monoclonal antibody, Thermo Fisher Scientific, Boston, MA, USA; clone SP6; pretreatment Tris95 C 30′ dilution: 1:200), phosphohistone H3 (PHH3; rabbit monoclonal antibody, Cell Signaling, Beverly, MA, USA; clone 5A1E; pretreatment Tris95 C 30′ dilution: 1:100) and cleaved Caspase 3 (rabbit monoclonal antibody, Cell Signaling, clone 5A1E; pretreatment: Tris95 C 30′ dilution: 1:100). Immunohistochemistry was performed on an automated platform (Leica Bond RX, Leica Biosystems, Muttenz, Switzerland).

#### Scoring of immunohistochemistry, mitotic figures and apoptotic bodies

For scoring, all slides were scanned to a digital platform, chronologically labeled and assessed by a blinded experimenter using Pannoramic viewer (3D Histech, Budapest, Hungary). The measurement tool was used to create annotation fields with a width of 300-500 *μ*m and a maximum depth of 200 *μ*m from the subcutaneous tissue to ensure scoring only in areas which received sufficient light power for ChR2(D156A) activation (see Figures 6a and c). Light propagation through the tumor was estimated by values known for blue light attenuation in brain tissue,^23^ the known light sensitivity of ChR2(D156A)^2^ and the assumption that the tight packaging and strong vascularization of the tumor leads to an additional light attenuation of approximately 25%. For each section, three annotation fields centrally and peripherally in the tumor containing an average of 469±171 cells each were scored (visual count, see Figures 6a and c). The percentage of positive cells was calculated. For immunohistochemical stainings, any staining intensity not attributable to background was considered positive. On the same platform, the corresponding areas were selected on H&E-stained slides for counting the percentage of mitotic figures and apoptotic bodies. Immunohistochemical stainings were scored by two independent observers and the H&E-stained slides were scored by a consultant pathologist.

### Illumination

A battery powered LED panel to hold a six-well cell culture plate was custom-designed in order to supply sustained pulsed illumination to ChR2(D156A)-YFP BLM within the cell incubator (see Figure 1c). The circular 5 mm LEDs (Conrad, Dietlikon, Schweiz; part number NSPB500AS Sel. wV/W, 470 nm, 11 000 mcd, 15) were arranged to supply consistent illumination 5 mW/mm^2^ across the whole cell culture plate.

LED-lining for mouse cages was custom-made by strips of interspersed high-power LEDs (Digi-Key electronics, Thief River Falls, MN, USA; part number XPEBLU-L1-R250-00Y01CT-ND). The 30 cm wide LED strips were placed on all four sides of the mouse cage just above the chaff (see Figure 6a) and consisted of three rows of single LEDs (475 nm, 33 lm, 130) with distances of 3 cm between LEDs in a row and the middle row aligned interspersed with the other two rows in order to achieve balanced illumination intensities throughout the cage. The subcutaneous light intensity above the tumor was estimated with a spectrophotometer (Thorlabs, Dortmund, Germany) held behind the skin of a sacrificed mouse and equaled 6.25 mW/mm^2^ for a mouse located in the center of the cage. Four hundred seventy-nanometer light was shown to have an attenuance of 0.2 in tumor tissue over a 0.2 mm path length.^22^ We therefore assumed the light intensity to reach saturating values for ChR2(D156A) activation to a tumor depth of minimally 5.8 mm^2^.

### Statistical analysis

Statistics was performed either by Excel or Graph Pad Prism statistics software (version 6.0.). For all normal distributions (verified using the Kolmogorov-Smirnov test), differences between optogenetically treated and control groups were analyzed using an unpaired one-tailed Student's *t*-test. All *in vitro* data (*n*⩽3) was analyzed using an unpaired two-tailed Student's *t*-test with Welch correction, not assuming equal variance. In the figures, different levels of significance are indicated by * if *P*<0.05, ** if *P*<0.01, if *** if *P*<0.001 and **** if *P*<0.0001. In the text, average values are indicated±standard deviation, unless otherwise indicated.

## Abbreviations

*ChR2*: *Channelrhodopsin-2*
*V*_*h*_: holding potential
PHH3: phosphohistone H3
YFP: yellow fluorescing protein
Ca^2+^: calcium
BAPTA: 1,2-bis(*o*-aminophenoxy)ethane-*N,N,N′,N′*-tetraacetic acid
CaCC: Ca^2+^-sensitive chloride channels
Ctrl: control
PI: propidium iodide
DMSO: dimethyl sulfoxide
MMP: mitochondrial transmembrane potential
H&E: hematoxylin and eosin
LED: light emitting diode
